# Use of Injection Five Fluorouracil (FFU) With or Without Injection Trimacinolone in the Management of Hypertrophic Scars and Keloids

**DOI:** 10.4103/0974-2077.41160

**Published:** 2008-01

**Authors:** Sharad Mutalik, Narendra Patwardhan

**Affiliations:** *Consultant Dermatologist, 562/5 Shivajinagar, Near Congress Bhavan, Pune, Maharashtra, India*

Sir,

Keloids are a common dermatological problem faced by Dermatologists and existing modalities of treatment are unsatisfactory. Though, different modalities have been tried, recurrences are common. We hereby report the efficacy of 5 fluorouracil (FFU) in treating keloids.

Thirty cases were selected for the study. Local anaesthesia was administered only for keloids >3 cm length. Field block anaesthesia was achieved by infiltrating injection bupivacaine 0.25% around the lesion. Injection FFU 50 mg/ml was infiltrated along the length of the keloid with a leur-lock syringe and 25G × 1½ needle. In cases of inflammed or hard keloids, injection triamcinolone 40 mg/ml was added to injection FFU in the ratio 1:1. Maximum dose of FFU used at a time was 1.5 ml.

Patient was sent home after the injection, with instruction to apply sodium fusidate or mupirocin ointment for 5 days. Non-steroidal anti-inflammatory drugs (NSAIDs) were advised for pain. Patients were assessed after 4 weeks and, the treatment was repeated if necessary.

Out of the 30 patients, six were lost to follow up. Sixteen patients showed complete flattening of keloids after an average of four injections. All these patients were advised to use Silicon-gel dressing for 3 months. Out of these 16 patients, 12 patients showed no recurrence at the end of 1 year.

Commonest side effect observed with FFU injection was temporary blackish discoloration in four cases. Infection was seen in one patient who received concomitant triamcenolone. Post-steroid hypopigmentation was seen in two cases.

Keloid is an overgrowth of dense fibrous tissue that extends beyond the borders of the original wound, whereas hypertrophic scar remains confined to the borders of the original wound. Abnormal wound healing results in disruption of the cascade of events in collagen synthesis, which leads to formation of hypertrophic scar or keloid.[1]

Exact aetiology of keloid is still not fully understood, but genetic predisposition is seen in cases of keloid, along with predisposition for anatomical sites like chest, shoulders, jaw line, upper back, gluteal region, ears, etc. Increased skin stretching at the above-mentioned anatomical sites and wound infection may be the factors contributing to the formation of keloids.

Usually treatment for keloids is sought for pain, pruritus, discomfort, cosmetic disfigurement and restriction of movements. Available treatment modalities include: intralesional steroid injections, cryotherapy with or without steroid injections, intralesional cryotherapy, excision followed by silicon-gel dressings, pulsed dye laser (585 nm), superficial X-ray therapy, etc. All these therapies have shown temporary regression in the size of keloids, but recurrence is known with all the modalities.[2]

Five fluorouracil has been shown to inhibit fibroblasts in tissue culture and is believed to reduce post-op scarring. Corticosteroids suppress inflammation and cause vasoconstriction. Their antimitotic effect results in inhibition of fibroblasts and keratinocytes.

In our study, complete flattening of keloid was seen in 75% cases with no recurrence. These preliminary results are encouraging, but further studies are needed to confirm these early findings.
